# A mini-review of aesthetic gynecology and leading gynecology associations’ approaches to this issue

**DOI:** 10.4274/tjod.33407

**Published:** 2018-06-21

**Authors:** Aylin Güneş, Red M. Alinsod

**Affiliations:** 1 Yeni Yüzyıl University Faculty of Medicine, Department of Obstetrics and Gynecology, İstanbul, Turkey; 2 South Cost Urogynecology, Clinic of Urogynecology and Pelvic Reconstructive Surgery, Laguna Beach, California, United States of America

**Keywords:** Aesthetic gynecology, labiaplasty, vaginoplasty, rejuvenation, radiofrequency

## Abstract

Aesthetic gynecology has seen increasing patient and physician demand. Although this typically falls in the reign of obstetrics and gynecology, plastic surgeons and cosmetic surgeons have also developed great interest in this field. Currently, few if any obstetrics and gynecology residency or fellowship programs teach this subject matter though inroads have taken place in plastic surgery and cosmetic surgery training programs that had the foresight to include specific training in this field. Currently, many surgeons start by first training in various established certification and preceptorship programs based in the United States and the United Kingdom. New programs worldwide in 2016-2017 have also been launched to offer certification training to interested physicians in both surgical and non-surgical treatments. A steady flow of certificate programs continues to evolve in Turkey, the Middle East, Spain, and South America, as a second wave of experts emerge. We present a review of surgical and non-surgical techniques of what is presently called “aesthetic gynecology” and the approaches of prominent gynecologic societies regarding this relatively new subspecialty.

## Introduction

Developments in both technology and fashion induce seasonal changes in the notion of beauty. The social and cultural differences among countries also play a highly significant role in this matter. Thus, one cannot give an exact description of the normal view of external genitalia. However, upon consideration of anatomic variations, Hodgkinson and Hait^(1) ^defined the ideal aesthetic picture of female external genitalia as the one in which the labia minora are small and not larger than the labia majora. The Motakef classification is based on the protrusion of the labia minora that exceeds the size of the labia majora^(2)^. The Banwell classification categorizes labia according to their shape and morphologic variations^(3)^. None of the classification systems have been accepted by gynecologic or plastic surgical societies and are rarely used. Apart from medical indications such as labial hypertrophy and congenital adrenal hyperplasia, most operations are performed upon the patient’s request due to a feeling of enlargement and looseness in the vagina, a desire to improve sexual function, discomfort when wearing clothes or doing fitness activities, or with an aim to increase sexual satisfaction for both herself and her partner. Regarding anxiety about external appearance, motives for surgery include perceived larger size or asymmetry of the labia minora and a dark-colored appearance of the labia majora^(4,5)^. Although it is not possible to define the ideal aesthetic genitalia, patient-specific techniques chosen based on the patient’s anatomy and applied with a realistic approach can increase patient satisfaction and reduce complication rates.

### Aesthetic gynecology surgical techniques

**Figure 1:**
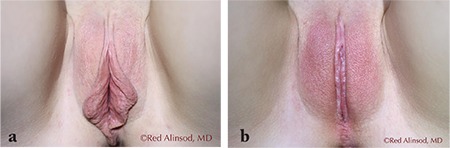
Young lady in her 20s felt uncomfortable with her labia and redundant and loose perineal tissues. She requested an aggressive labiaplasty for both comfort and personal confidence a) before surgery, b) two months after surgery (labia minoraplasty, clitoral hood reduction, and perineoplasty)

Vaginal tightening, or vaginoplasty, refers to surgery of the vaginal entrance, deeper canal, and epithelium. This procedure is not the same as pelvic floor repair and if there is pelvic floor defect it should be part of urogynecologic assessment. Frequently, it is considered as a modification of standard posterior repair but may include excision of lateral vaginal mucosa or high posterior repair^(6,7)^. It is usually carried out along with perineoplasty and paravaginal repair,with or without an anterior colporrhaphy,to adress patient concerns of having a large or loose vagina. From the perspective of cosmetic gynecology, the surgeon must determine the limits of the planned vaginal diameter reduction, in advance, in dialogue with the patient, and keep in mind that the patient’s expectation might be unrealistic. The risks of over-tightening must be explained. Perineoplasty is a specific repair of the vaginal entrance and the space between the vaginal and the anal openings. It is a complementary procedure to prolapse surgery. The surgical goals are cosmetic achievement through reformation of the perineal body, thereby lifting the perineum, and greater sexual satisfaction through increased friction with penile penetration. Also it can straighten out the path that stool passes through and improve defecation mechanics. However, when perineoplasty is applied alone to ameliorate sexual dysfunction, conclusive results indicating successful application are lacking^(4)^. Labiaplasty is the most commonly applied aesthetic genital surgery and should be aimed at improving the appearance of the external genitalia and reducing obstructive tissues during intercourse. The surgical reduction of the labia minora was first described as an aesthetic motive in the plastic surgery literature by Hodgkinson and Hait^(1)^. When we look at the literature, many techniques have been described for labiaplasty: deepithelialized reduction, linear incision, composite reduction, wedge reduction, W-plasty excision or Z-plasty^(2)^. The linear excision technique is the most preferred among gynecologists because of its simple and minimally invasive approach. In Laguna Beach, California, terms that are used for the labial reductions according to the level of labia majora below or at the same level with labium minora: rim look, Barbie look or hybrid look^(8)^. With this technique, smoother contours can be achieved. According to Miklos and Moore’s^(5)^ study, 97% of 550 women wanted to remove dark edges. If the patient wants to retain the dark edges of the labium minora, a wedge technique can be performed. This technique is mostly performed by plastic surgeons. There are modifications of this technique; central V-plasty, and 90-degree Z-plasty^(9,10,11)^. If any of these wedge techniques are performed, perfect hemostasis is very important to prevent wound dehiscence and fistula formation. Although the wedge resection technique preserves a more natural edge look, most women want removal of this irregular labial edge for a smoother and more petite appearance (Figure 1a, b). If a minimal amount of labium needs to be excised deepithelialized reduction technique may be preferred^(12)^. If the deepithelialized area is large, it can result in increased labial thickness and a visible suture line^(13)^. Labia majora reduction, or labia majoraplasty, is intended to reduce the size of labia majora that appear saggy and hyperplastic. Here, the excess skin, and, if necessary, fat pad is removed. In over 90% of cases, only segments of the labia majora skin is removed. Longitudinal resection with scar placement between the thigh-vulva crease or resection with scar placement between the labium majora and minora can be performed. The first technique has high risk for wound dehiscence because of scar formation in a high tension area^(14)^. 

**Figure 2:**
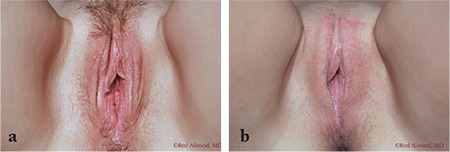
Middle-aged multiparous female, unhappy with the appearance of her loose labia majora and her introitus. She requested a labia majoraplasty and perineoplasty and declined vulvar filling procedures a) before labia majoraplasty: front view, b) months after labia majoraplasty: front view

The labia majoraplasty procedure is employed to address medical conditions such as congenital lymphedema and sagginess from chronic steroid use in conditions such as congenital adrenal hyperplasia. When performed for aesthetic concerns, labia minoraplasty can be performed in conjunction with labia majoraplasty, and are typically separate procedures (Figure 2a, b). Clitoral hood reduction (hoodoplasty) is performed with labia minoraplasty, but not always. One of the techniques is to reduce the clitoral hood skin over the clitoris using the skinning deepithelialization technique, lateral in location. A vertical clitoral hood reduction technique is performed for wide hoods and redundant hoods with multiple skin folds. The composite reduction technique is a type of minoraplasty and it also achieves clitoral protrusion correction^(15)^. The clitoris is not unhooded in the large majority of cases and the procedure is performed to achieve better symmetry and reduce the “top-heavy” look post labia minoraplasty. Goodman^(7)^ defined the goal of this reduction as improving sexual arousal by revealing more of the clitoris. 

Labia Majora augmentation is not considered to be a surgical technique, its purpose is to aesthetically improve labia majora with a hypoplastic or loose appearance. To this end, autologous fat-grafting or hyaluronic acid (HA) fillers are mostly used. Although the region selected for autologous fat grafting may be any area with abundant fatty tissue, the most frequently used regions are the thigh and the inner part of the knee. The fat graft is prepared through techniques such as a washing centrifuge; the Coleman^(16)^ technique is the most frequently used. For an autologous fat graft, the injection must take into account re-absorption, otherwise a second session may be required to achieve the desired result. The use of HA as a filler is also a frequently employed technique in non-surgical rejuvenation. Fat is the predominant product used in the United States of America (USA) and HA is predominant in Europe due to costs. In the USA, filling of the labia majora is not as popular as in Europe. The preference for American women is a sleeker and more petite appearance, which labia majoraplasty or radiofrequency (RF) shrinkage can help achieve. In Europe, there is more use of filling techniques, specifically HA. The outcome is determined by the surgeon’s anatomic knowledge and operating skills. However, the use of HA in this region must be carefully considered. Proper techniques and materials can prevent inflammatory developments, such as a granuloma formation.

Hymenoplasty involves many ethical issues. It is known as revirgination in Western countries and is a socio-cultural issue, especially in Muslim countries. The operation itself is the least-studied female genital procedure. It is most accurate to classify hymenoplasty as a reconstructive procedure and exclude it from the set of aesthetic procedures. The ethics committees of many associations do not consider it as cosmetic genital surgery^(17,18)^. This surgery can be life-saving for women. It is a simple procedure and can be described as a “vaginal repair” for the patient’s protection of privacy.

### Aesthetic gynecology non-surgical techniques

**Laser treatment for vaginal laxity:** several fractional lasers have been used for non-invasive treatment of vaginal laxity. Fractional carbon dioxide (CO_2_) lasers emit light at a wavelength of a 10.600 nm, which is strongly absorbed by tissue water. The penetration depth is dependent upon the water content, independent of melanin and hemoglobin. It stimulates and promotes the regeneration of collagen fibers and restores hydration and elasticity in the vaginal mucosa^(19)^. Fractional erbium laser is a minimally invasive thermo-ablative fractional laser technique, which is applied to vaginal mucosa and is used in postmenopausal vulvar-vaginal atrophy, stress urinary incontinence, and vaginal tightening. With its wavelength of 2940 nm, it is close to the absorption peak of water. This laser has 10 to 15 times more affinity for water absorption compared with the fractional CO_2_ laser. The photothermal effect of the laser beam heats the collagen in selected mucosal tissue leading to the contraction of collagen fibers and at the end shrinkage of tissue. It has minimal thermal damage to surrounding tissue so has milder postoperative discomfort and edema^(20)^. Laser vaginal rejuvenation was trademarked by Dr. David Matlock. It is performed with a 980 nm diode laser used as a cutting device, much like standard cautery, and not in the newer minimally invasive fractional manner now used by many laser companies to shrink the vaginal walls.

**Figure 3:**
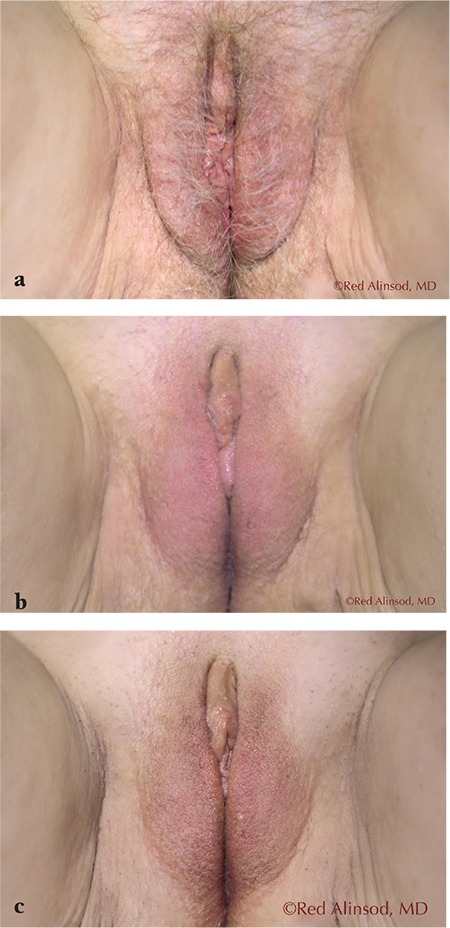
A 66-year-old female with severe genitourinary syndrome of menopause and labia majora laxity underwent monthly transcutaneous temperature-controlled radiofrequency vaginal rejuvenation x6 to achieve maximum labia majora shrinkage without surgery. Shown is the progressive tightening effects obtained over the 6-month period. Internal treatments were also done that enabled comfortable sex to be possible without the need for added lubricants a) before treatment, b) after 3 treatments, c) after 6 treatments

**Figure 4:**
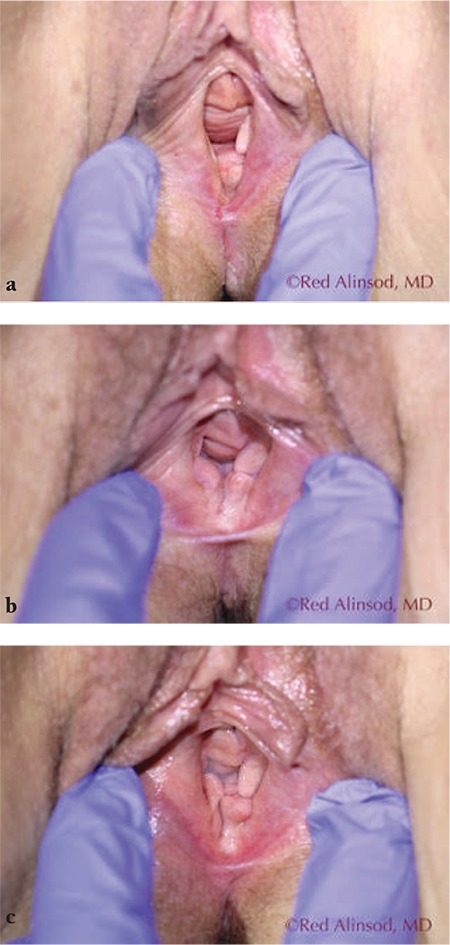
A 60-year-old female with severe genitourinary syndrome of menopause and tearing of perineum during intercourse. She underwent transcutaneous temperature-controlled radiofrequency monthly for 3 sessions and obtained excellent relief from the dryness and dyspareunia. Her incidentally-found mild cystocele was also reduced in size a) before treatment, b) after 2 treatments, c) after 3 treatments

**RF vaginal rejuvenation:** this energy-based skin rejuvenation technology has been harnessed for rejuvenation of vaginal tissue to treat vulvovaginal laxity resulting from age or childbirth-related causes. Studies have shown that the use of RF for vulvovaginal laxity results in increased collagen and elastin formation^(21)^. Unlike laser-based treatments, it is not dependent on skin type and is even more effective in naturally moist tissue. This technique has been demonstrated to be especially well tolerated when using temperature-controlled RF. The target tissue temperature is 40-45 degrees Celsius, and thermistors enable monitoring and thermostating the temperature. This technique enables collagen denaturation and the healing process, supporting healthy tissue formation, which is the mechanism that provides tightening. Collagen fibers when heated contract and this causes the triple helix structure to fold, creating thicker and shorter collagen fibers, which are thought to be the mechanism of action of the immediate tissue tightening seen after these procedures (Figure 3a, 3b, 3c). The creation of new elastin, which is relatively unique to RF, may play a role in its effectiveness in treating vaginal laxity^(22)^. Additionally, it has been found that the increased local blood flow with this technology leads to decreased dryness due to vulvovaginal atrophy, resulting in improved sexual performance and satisfaction (Figure 4a, b, c)^(23,24)^. Researchers have also shown regression of stress urinary incontinence with tightening of the pubocervical fascia^(25,26)^.

**Vulvar lightening:** this technique achieves whitening of a hyperpigmented vulvar appearance through chemical agents or the CO_2_ fractional laser method. Avoiding rebound hypo-hyperpigmentation should be the prime objective^(27)^. Hyper and hypo-pigmentation can occur with the use of energy-based devices such as a CO_2_ laser to lighten the area. The use of RF in an ablative manner can also result in both hypo or hyperpigmentation. Non-ablative RF avoids these pigment issues.

**Platelet-rich plasma (PRP):** autologous PRP was first reported in 1987 for open heart surgery^(28)^. Over 20 years it has been studied in wound care, orthopedics, dental surgery, spine literature, and a variety of cosmetic surgery procedures. PRP contains high level of growth factors such as platelet-derived growth factor, transforming growth factor beta and epidermal growth factor. It is nonantigenic because it is autologous, and there have been no detected adverse effects^(29)^. It has been found that PRP injections are nonsurgical options for female sexual dysfunction, lack of lubrication, and stress urinary incontinence^(30)^. Some pilot studies have also shown that PRP has an effect in the treatment of lichen sclerosis^(31,32)^. O-Shot^®^ is a PRP procedure that was trademarked by Charles Runels.

The combination of PRP with RF for lichen sclerosis has shown tremendous promise for long term symptoms relief. Studies in the United States with Dr. Runels and Dr. Alinsod are ongoing. augmentation: In 1950, German gynecologist Grafenberg^(33)^ described an erotic zone on the anterior vaginal wall along the course of urethra. Since then, many articles have been published showing the existence of this zone and in 1981 this area was named as the G-spot by Addiego et al.^(34)^ to honor Grafenberg^(33)^. Many authors accept this area as the responsible zone for vaginally-activated orgasm. The precise anatomy is not fully understood but can be defined as a neurovascular complex^(35,36)^. G-spot augmentation with fillers such as collagen or autologous fat transplantation leads to bulking of this zone to the vaginal lumen and much more penetration during sexual intercourse^(37)^.

## Discussion

The perception of ideal external female genitalia or ideal labium appearance differs between countries^(38)^. The desired appearance according to countries affects the surgeons’ techniques^(8)^. According to the American Society for Aesthetic Plastic Surgery, labiaplasty numbers increased 23% from 2015 to 2016^(39)^. Various techniques may be applied in labia minoraplasty according to the state, color change, and expectations of hypertrophy.  Any desired reduction of labia can be provided with linear excisions but it cannot provide the retention of the natural look or coloration the patient currently has. The wedge resection and modifications of this technique may serve for patients who want more natural edges. When performed by surgeons trained and experienced in this field, these operations are demonstrated to improve the reliability of the procedures and the functional and aesthetic appearance^(8)^.

Nonsurgical techniques like transcutaneous temperature-controlled and laser devices are also options for aesthetic genital interventions, especially for vulvovaginal laxity. Studies also showed some changes in vulvovaginal atrophy and stress urinary incontinence^(40)^. Patients with severe organ prolapse are not candidates for nonsurgical aesthetic techniques. Therefore, a careful examination should be performed to evaluate pelvic organ prolapse and several self-reported questionnaires can be used to assess the degree of symptoms^(41)^. There are some controversial issues as to which energy-based device is superior (laser or RF) or which type of laser device should be preferred. Unlike surgical techniques, non-surgical approaches need maintenance treatments, and treatment protocol differ according to the device. For the standardization of treatment modalities and an explanation of duration of efficacy we need further research.

Let us briefly examine how the world’s leading gynecologic associations evaluate this issue. When the American Collages of Obstetricians and Gynecologist (ACOG) first addressed this issue in 2007, it opined that procedures such as vaginal rejuvenation designer vaginoplasty, and revirgination were seperate from procedures with non-aesthetic medical indications which had insufficient research so far^(17)^. However, after this committee’s declaration, Ostrzenski.^(42)^, in 2011, published extensive evidence-based work on the effectiveness and reliability of these procedures and concluded that the ACOG’s 2007 recommendations did not comply with scientific norms and were not sufficiently transparent. Iglesia^(43)^ in 2012, emphasized that the term “perfect vagina” represented a significant domain in the concept of women’s beauty and that doctors must inform their patients about the complications and all the relevant details concerning this matter. In 2013, Canada’s Society of Obstetricians and Gynaecologists’s policy statement recommended that the medical, sexual, and gynecologic histories be reviewed with patients requesting genital cosmetic surgery.  They also recommended that the patient be informed of the normal variations in genital appearance, the physiologic changes that develope with aging, and the unpredictability of changes that might occur during pregnancy and menopause^(44)^. In 2013, the Royal College of Obstetricians and Gynaecologists published ethical considerations in relation to female genital cosmetic surgery and recommended that “female genital cosmetic surgeries shouldn’t be carried out before 18 years of age, the patient must be fully concerned about the procedures, and any advertising of these procedures conforms to good medical practice”^(45)^. In 2015, the International Federation of Gynecology and Obstetrics Committee for the Ethical Aspects of Human Reproduction published a report supporting that patients requesting cosmetic gynecologic procedures and surgeons must be aware of the differences between therapeutic surgical procedures and surgical procedures without medical indications, that normal anatomy and variations must be explained so that patients have a good understanding of them, that patients should be evaluated, especially for body dysmorphic disorder and other mental problems, and that the operating surgeons must have competent skills in this field^(46)^.

In 2017 ACOG published a new committee statement recommending that in the case of requests for mammoplasty and labiaplasty, patients, especially adolescents, and their families be informed about normal variations and physical changes, that the patient’s physical and emotional development had to be evaluated, and that consultation about non-surgical techniques should be provided^(47)^.

According to the World Health Organization, the definition of female genital mutilation refers to all procedures involving partial or total removal of external genitalia^(48)^. It has no health benefit and it is a human rights violation. However, this description is totally different from female genital cosmetic surgery relating to genital destruction and the lack of patient consent in mutilation.

In conclusion, the lines between medically necessary operations such as vaginal/pelvic reconstructive surgery and elective surgeries such as vaginoplasty and labiaplasty are blurring and can now be performed at the same time. Both function and beauty are becoming addressed together and not separately. Minimal complication rates and maximum patient satisfaction can be achieved if experienced, trained physicians are involved. To achieve the best outcomes in both functionality and appearance, surgeons must inform patients about normal variations, perform psychological evaluations, and discuss realistic expectations. The technique of surgery to be used should be individualized with great consideration of the patient’s preferred goals and with realistic expectations.  The surgeon’s training and skills and comfort level with the various techniques must be considered fully. As is clearly seen, more academic training in this branch of gynecology must be given. Moreover, further studies are needed on the long-term efficacy, safety and reliability of non-surgical techniques, especially those that do not require hospitalization and can be performed in an office environment.
